# Debate around infection-dependent hemophagocytic syndrome in paediatrics

**DOI:** 10.1186/1471-2334-13-15

**Published:** 2013-01-16

**Authors:** Valentina Ansuini, Donato Rigante, Susanna Esposito

**Affiliations:** 1 Pediatric Clinic 1, Department of Pathophysiology and Transplantation, Università degli Studi di Milano, Fondazione IRCCS Ca’ Granda Ospedale Maggiore Policlinico, Via Commenda 9, 20122, Milan, Italy; 2 Institute of Pediatrics, Università Cattolica Sacro Cuore, Rome, Italy

## Abstract

**Background:**

Hemophagocytic syndrome (HPS) is clinically defined as a combination of fever, liver dysfunction, coagulation abnormalities, pancytopenia, progressive macrophage proliferation throughout the reticuloendothelial system, and cytokine over-production, and may be primary or secondary to infectious, auto-immune, and tumoral diseases. The most consistent association is with viral infections but, as it is still debated whether any micro-organisms are involved in its pathogenesis, we critically appraised the literature concerning HPS and its relationship with infections.

**Discussion:**

Infection-dependent HPS has been widely observed, but there are no data concerning its incidence in children. A better understanding of the pathophysiology of HPS may clarify the interactions between the immune system and the variously implicated potential infectious agents. Epstein-Barr virus (EBV) infection has been prominently associated with HPS, with clonal proliferation and the hyperactivation of EBV-infected T cells. However, a number of other viral, bacterial, fungal, and parasitic infections have been reported in association with HPS. In the case of low-risk HPS, corticosteroids and/or intravenous immunoglobulin or cyclosporine A may be sufficient to control the biological process, but etoposide is recommended as a means of reversing infection-dependent lymphohistiocytic dysregulation in high-risk cases.

**Summary:**

HPS is a potential complication of various infections. A polymerase chain reaction search for infectious agents including EBV, cytomegalovirus and *Leishmania* is recommended in clinical settings characterised by non-remitting fever, organomegaly, cytopenia and hyperferritinemia.

## Background

Hemophagocytic syndrome (HPS) is a potentially fatal condition due to dysregulated lymphocyte activation and proliferation, mainly characterised by impaired or inactive natural killer (NK) cells and cytotoxic T cells, which leads to macrophage hyperactivation and over-expression of cytokines [1]. The result of this process is uncontrolled and ineffective immune activation, multi-organ dysfunction, and hemophagocytosis throughout the reticuloendothelial system [2]. The pathognomonic characteristic of HPS is the activation of well-differentiated macrophages, phagocyting erythrocytes, leukocytes and platelets in bone marrow, lymph nodes, spleen, liver and other organs, which can infiltrate almost anybody district and may account for many of its systemic features [3]. HSP is still often under-diagnosed and sub-optimally managed in children [4], but the epidemiological data are fragmentary.

The syndrome was first described in 1939 as poorly-controlled histiocyte proliferation, but has since also been called hemophagocytic histiocytosis and macrophage activation syndrome [5-7]. It can be divided into a primary genetic form and a secondary reactive form (Table 1), a distinction that has historically been used to differentiate cases of often fatal infantile HPS from those caused by other etiologies that appear later in life and have a better prognosis. This difference may be artificially scholastic because primary forms can occur at any age (not only during infancy or early childhood) [8], and both primary and secondary forms can be precipitated by infections with a substantial risk of mortality [9]. Even secondary HPS occurs as an imbalance between insufficient host defense, obstinate hyperinflammation, and a heterogeneous triggering event, which can be of infectious, rheumatic or neoplastic nature: therefore, the clinical disease results as the signature of a dysregulated immune activation, leading to macrophage proliferation and widespread hemophagocytosis in the reticuloendothelial system. The aim of this review is to make a critical appraisal of the literature concerning infection-related HPS in paediatrics.

**Table 1:** Classification of hemophagocytic syndrome

**Type of hemophagocytic syndrome**	**Subtype**
**Primary or genetic hemophagocytic syndrome**	Familial hemophagocytic lymphohistiocytosis
	Immune deficiency syndromes (i.e., Chediak-Higashi syndrome, Griscelli syndrome, X-linked lymphoproliferative syndrome)
**Secondary or reactive hemophagocytic syndrome**	Infections (i.e., viral, bacterial, parasitic)
	Malignancies
	Auto-immune and auto-inflammatory diseases

## Discussion

### A multi-face disease with variable causes

Genetic HPS is heterogeneous and arises from defects in the critical regulatory pathways responsible for the natural termination of immune responses that lead to the failure of the homeostatic removal of cells that are superfluous or dangerous to the host. Since 1999, various genetic loci related to the activity of perforin and granzyme granules have been associated with genetic autosomal recessive HPS, thus explaining the impaired or absent function of NK cells and cytotoxic T cells [2,6]. The familial form, which was first described in 1952 [10], is an autosomal recessive disorder that is estimated to occur in 1/30,000-50,000 births, and usually appears during the first year of life [11,12]. Sporadic cases of HPS associated with multiple genetic mutations have also been described [13]. The different primary forms of HPS are associated with immune deficiencies, including Chediak-Higashi syndrome, Griscelli syndrome, X-linked lymphoproliferative syndrome, Wiskott-Aldrich syndrome, severe combined immunodeficiency, lysinuric protein intolerance, and Hermansky-Pudlak syndrome. Acquired HPS has also been associated with a variety of viral, bacterial and mycobacterial, fungal, and parasitic infections [14], auto-immune diseases [15], auto-inflammatory syndromes [16], and tumours, particularly T cell malignancies [17].

The constellation of signs and symptoms of HPS is not specific and none of the biochemical abnormalities is distinctive. The dramatic presentation of the syndrome includes unremitting fever, visceromegaly, thrombocytopenia, lethargy, seizures, skin rash, pulmonary failure, and cardiac and/or renal involvement, and the mortality rate is 8-22% [18]. The most common laboratory findings are due to liver dysfunction, and include low fibrinogen, and high serum triglycerides and ferritin levels [18]. Two highly diagnostic clues are increased plasma concentrations of the alpha chain of interleukin-2 receptors (also known as sCD25) and impaired NK cell activity. As treatment can be life-saving and some of the clinical criteria occur late during the course of the disease, it is not necessary to satisfy all of the criteria before beginning therapy. The hallmark of HPS is the phagocytosis of blood cells and their precursors: bone marrow aspiration typically reveals the normal maturity of all cell lineages, and infiltration by activated macrophages “stuffed” with other blood cells [19].

Criteria for a diagnosis of HPS on the basis of clinical, laboratory, and histopatological findings are the following [3]:

(1) Genetic diagnosis: genes known to cause the syndrome (*PRF1, UNC13D, STX11, STXBP2, RAB27A*)

(2) Signs and symptoms (at least five of the following criteria):

a) Fever

b) Splenomegaly

c) cytopenias (minimum 2 cell lines reduced)

d) hypertriglyceridemia (≥ 265 mg/dL) and/or hypofibrinogenemia (≤ 150 g/dL)

e) hemophagocytosis in any involved organ

f) very little or no NK cell activity

g) increased ferritin ≥ 500 mg/L

h) increased soluble CD25 (serum interleukin-2 receptor alpha) ≥ 2.400 U/mL

The main pathophysiological abnormality in HPS is cytokine dysfunction, which leads to the uncontrolled accumulation and ectopic migration of activated T lymphocytes, antigen-presenting cells and histiocytes, and multi-system inflammation [20]. The pathophysiology of acquired HPS has not been fully defined, but deficient cytolytic activity leads to the persistent activation of lymphocytes and histiocytes, followed by the hypersecretion of pro-inflamatory cytokines and high soluble interleukin-2 receptor levels that correlate with the prognosis [21].

### Infectious etiologies of HPS

The association between HPS and infections has been widely documented and both familial or sporadic cases are often precipitated by acute infections. It must also be pointed out that every form of HPS can mimic infectious diseases or overwhelming bacterial sepsis, thus hindering the diagnosis of a precipitating and treatable infectious illness. Virus-associated HPS was first described in 1979 by Risdall *et al*., whose series consisted of 19 patients, most of whom were immunocompromised but without any confirmed genetic or acquired immunodeficiency, and all of whom showed serological signs of viral infection [22,23]. Since then, there have been reports of HPS associated with a host of infections [22].

#### HPS associated with viral infections

Epstein-Barr virus (EBV) is the most commonly reported trigger of HPS [24]. The epidemiology of EBV-related HPS is not well known, although a higher incidence has been observed in Asian countries, where it has been theorised there may be a more pathogenic viral strain that is genetically similar to the strains observed in nasopharyngeal carcinoma cell lines [25]. Two forms of EBV-related HPS have been described: the first occurring during primary infection and the second during a reactivation process [26]. During primary infection, EBV typically infects and replicates in B cells, whereas EBV-specific cytotoxic T cells are required to produce memory cells. In rare cases, EBV may infect T and NK cells and induce persistent EBV infection, which may lead to chronic active EBV infection, lymphoproliferative disorders and fulminant EBV-related HPS [27-29]. Serological testing can help determine whether EBV-associated HPS has occurred in the setting of acute infection or is the result of a reactivation process. In addition, the real-time polymerase chain reaction (PCR) detection and quantification of EBV nucleic acid is an important laboratory means of adequately reflecting viral replication and assessing EBV load in patients with EBV-related HPS [30]. The quantitative analysis of cell-free EBV genome copy numbers after four months of treatment can assess therapeutic responses and is prognostically significant [31].

The clonal expansion of EBV-infected T lymphocytes has been demonstrated in EBV-related HPS [32] and EBV-positive T cell lymphoma [33] on the basis of the presence of homogeneous viral terminal repeat sequences. The clonality of infected T lymphocytes is further suggested by the finding of monoclonal rearrangements of the T cell receptor-alpha gene in EBV-related HPS [34]. The distinction between the monoclonal proliferation of T lymphocytes seen in EBV-related HPS and EBV-positive T cell lymphomas may describe the extremes of a spectrum of disordered T lymphocyte proliferation following EBV infection. The inflammatory cytokine over-production seen in patients with EBV-related HPS tends to be much more pronounced than that observed in patients with other forms of HPS [35]. Of all of the infections associated with HPS, EBV infection has the worst prognosis in the presence of underlying hereditary disorders, diffuse intravascular coagulation, neutropenia, or central nervous system involvement [36].

Treatment strategies vary significantly depending on the clinical features of the infection: mild cases of EBV-related HPS are treated conservatively as spontaneous regression has been reported, and antiviral therapy with acyclovir, ganciclovir or cidofovir has led to disappointing results [37]. In the case of severe EBV-related HPS, the introduction of immuno-chemotherapy and, if necessary, allogenic stem cell transplantation has radically changed the history and prognosis of the disease: in such cases, the optimal treatment strategy can be centred on immunosuppressive medications that inhibit overactive T and NK cell responses (i.e. corticosteroids, cyclosporine A, intravenous immunoglobulin, anti-thymocyte globulins, etoposide, rituximab, and plasma or blood exchange transfusions) [38,39]. Hematopoietic stem cell transplantation is the last treatment resort for refractory forms of EBV-related HPS, and in the case of EBV infection occurring in genetic forms of HPS [40].

The most frequent herpes viruses associated with HPS other than EBV are cytomegalovirus (CMV) and human herpes virus 8 (HHV8).

CMV up-regulates tumour necrosis factor gene expression and has been associated with HPS in otherwise healthy patients, patients with inflammatory bowel disease, rheumatological diseases and cancer, and transplant recipients [41]. HPS was observed in seven of a series of 171 patients undergoing hematopoietic stem cell transplantation, and was triggered by CMV in three cases [42]. Younger age may be associated with a worse prognosis [43]. A recent study has shown that the use of specific anti-CMV therapy, such as CMV immunoglobulin, foscarnet or ganciclovir, may be therapeutic [44].

HHV8 has been associated with HPS in 13 patients: most of these cases occurred in patients with a lymphoproliferative disorder [45] or immunocompromised patients [46], and rarely in immunocompetent hosts [47]. Treatment based on etoposide, ganciclovir, foscarnet or rituximab has led o successful results [48].

HPS can be associated with human immunodeficiency virus (HIV) infection, alone or with a wide variety of underlying disorders. It is likely that this condition is underestimated as HIV infection and HPS have many clinical and laboratory similarities. About 10% of bone marrow biopsies taken from HIV patients before the start of highly active antiretroviral therapy show active signs of hemophagocytosis [49]. HIV-related HPS can be observed in cases of acute or late HIV infection, and in conjunction with immune reconstitution inflammatory syndrome, opportunistic infections, or malignancies [50]. HPS may even be the initial presentation of HIV infection [51], and it has been suggested that HIV itself may play a direct role in triggering the syndrome [52]. Other common viral triggers of HPS in HIV patients are EBV, CMV and HHV8, and EBV-related HPS seems to be more frequent in HIV-infected children [53].

Influenza-related HPS has been rarely reported in immunocompromised and otherwise healthy children [54-57]. One fatal case of HPS was observed among 32 children hospitalised with seasonal influenza in a prospective pediatric study [58], but reactive HPS has also been associated with avian and swine (non-pandemic) influenza [59,60]. In particular, patients with severe H5N1 (avian) influenza infection have symptoms and laboratory findings that are similar to those observed in patients with HPS, mainly encephalitis, organ dysfunction with hemophagocytosis, bone marrow failure, and pro-inflammatory cytokine over-production [61]. Clinical studies have found that mutations in some viral genes (NS1, PB2, HA and NA) are significantly related to cytokine release, and it has been demonstrated that recombinant hemagglutinin (H5) from H5N1 virus can suppress perforin expression and reduce the cytotoxicity of T cells, including their ability to kill H5-bearing cells [62]. Some authors have suggested treatment with a shorter course of etoposide and dexamethasone [63].

HPS has been reported in 28 cases of parvovirus B19 infection, most of whom had hereditary spherocytosis as the underlying disease: fewer than half were children [64-68]. Of these patients, 16 did not receive any treatment and 22 survived, thus suggesting that the prognosis of parvovirus-associated HPS is better than that of the other viral-mediated forms of HPS.

Fulminant viral hepatitis may mimic and even cause HPS, with hepatitis A virus being more frequently associated with HPS than the other hepatotrope viruses. Fifteen cases (including children) have been described, mainly in Asia: three of these patients also had a concurrent rheumatological disease (systemic juvenile idiopathic arthritis or Still’s disease) and two also had hepatitis C. Their treatment consisted of corticosteroids, variously combined with intravenous immunoglobulin, but four patients received no specific treatment and 11 of the 15 experienced a favourable outcome [69-71].

Enterovirus-related HPS has been described in 12 pediatric cases: five occurred in infants aged <1 year, and the oldest patient was 18 years old. An underlying disease was found in four patients who experienced a fatal outcome (lymphoid neoplasms, lymphoblastic leukemia and juvenile idiopathic arthritis). Ten patients received intravenous immunoglobulin (six in combination with corticosteroids), but only seven patients survived [72].

Other viruses associated with HPS include adenovirus, paramyxovirus (leading to measles and mumps), rubella virus, human parainfluenza viruses, *Flavivirus* (leading to dengue fever) and hantavirus (leading to hemorrhagic fever and severe acute respiratory syndrome), all of which have been treated with varying courses of corticosteroids and intravenous immunoglobulin.

#### HPS associated with bacterial infections

Reactive HPS has frequently been associated with intracellular pathogens. The pathophysiology of HPS associated with non-viral agents may be related to the production of high levels of activating cytokines by host lymphocytes and monocytes. Although the pathophysiological response of the host immune system to the infectious agent is not fully understood, it is hypothesised that functional deficiencies in NK and cytoxic T cells may occur during the illness [73].

HPS can be associated with disseminated *Mycobacterium tuberculosis* infection. Thirty-six cases (including infants and children) have so far been reported, approximately half of which were accompanied by comorbidities: eight patients had end-stage renal disease and were receiving hemodialysis or had undergone renal transplantation, four had a history of a malignancy, two had AIDS, and one had sarcoidosis. Fever was the most frequent clinical feature upon presentation, combined with visceromegaly and pancytopenia, and all of the patients underwent bone marrow aspirations that confirmed hemophagocytosis. Evidence of extra-pulmonary tuberculosis was found in 83% of cases. The concluding remarks of the report stated that tuberculosis-related HPS has a poor prognosis, with a mortality rate of approximately 50%, although anti-tuberculous and immunomodulatory therapy (consisting of high-dose corticosteroids, intravenous immunoglobulin, anti-thymocyte globulin, cyclosporine A, epipodophyllotoxin or plasma exchange) may lead to a better outcome [74]. Early diagnostic confirmation and the timely administration of anti-tuberculous medication seem to be crucial in these patients. One reported case of HPS occurred after childhood vaccination with the bacillus Calmette-Guérin [75].

HPS has also been described in association with brucellosis, with *Brucella melitensis* being the most frequently isolated organism [76]. Leptospirosis can cause life-threatening HPS as a result of an insufficient or misdirected immunological response to *Leptospira* itself: antibiotic treatment alone is not sufficient in such cases, and treatment with corticosteroids, intravenous immunoglobulin or etoposide is required [77]. Rickettsial diseases, transmitted to humans by arthropod bites and usually controlled at an intracellular level by nitric oxide synthesis, hydrogen peroxide production, and tryptophan degradation have also been related to HPS: overall, 15 cases of rickettsial disease confirmed serologically and complicated by HPS have been published in the period 1990–2010, with only 3 cases occurring in patients less than 15 years and a prognosis influenced by the specific *Rickettsia* species, patient’s immunologic equipment, and delay in antibiotic therapy or corticosteroid therapy [78]. In 2009, sepsis caused by multidrug-resistant *Acinetobacter baumannii* following urinary tract infection was reported for the first time in a previously healthy 3-year-old child, who recovered after multiple doses of granulocyte colony stimulating factor and red blood cell/platelet transfusions without any cytotoxic treatment or immunotherapy [79].

#### HPS associated with parasitic and fungal infections

HPS can be associated with *Leishmania donovani* and *Leishmania infantum* infections, but leishmaniasis may also mimic the syndrome, as it is characteristically associated with organomegaly and pancytopenia. This is particularly important in non-endemic areas, where visceral leishmaniasis is unlikely to be included in the differential diagnosis, and repeated bone marrow smears are often required to identify *Leishmania* species by means of PCR with species-specific probes [80]. Specific anti-leishmania treatment with amphotericin B is usually sufficient to control HPS. Unfortunately, sporadic cases of undiagnosed leishmaniasis have been treated as HPS with fatal consequences [81].

Malaria (caused by *Plasmodium falciparum* and *Plasmodium vivax*), toxoplasmosis, babesiosis, and strongyloidiasis have been rarely identified in association with HPS: a history of travel from endemic countries may help to identify these triggering agents [82].

Yeast (*Candida* spp., *Cryptococcus* spp. and *Pneumocystis* spp.) and moulds (*Histoplasma* spp., *Aspergillus* spp. and *Fusarium* spp.) have been associated with the occurrence of HPS, most commonly during HIV infection, neoplastic diseases, protracted corticosteroid administration, and transplantation [83-85].

Disseminated *Penicillium marneffei* infection is common among HIV-infected patients in many regions in Southeast Asia: the first case of HPS associated with penicilliosis in a Thai HIV-infected child was reported in 2001, with complete recovery after antifungal and intravenous immunoglobulin therapy [86].

## Conclusions

Many immunological, neoplastic and genetic disorders may underlie HPS, but infectious causes are the most prevalent and most frequently reported in association with this syndrome.

The specific clinical and laboratory tests for microbiological identification of HPS are the following:

(1) Blood, mid-stream urine, cerebrospinal fluid and sputum cultures for bacteria and fungi; cultures/microscopy for mycobacteria; tuberculin skin test and/or interferon-release tests

(2) Bone marrow aspirate

(3) Serological tests for acute Epstein-Barr, cytomegalovirus and human immunodeficiency virus infections

(4) Peripheral blood nucleic acid tests for evidence of replicating Epstein-Barr virus, cytomegalovirus, herpes simplex virus, human herpes virus-8, human immunodeficiency virus, adenovirus and parvovirus B19

(5) Serological tests for evidence of primary toxoplasmosis or reactivation

(6) *Leishmania* antigen test

(7) Serum cryptococcal antigen and serum galactomannans

As a fatal outcome may occur when infection-related HPS is only treated supportively, a multidisciplinary approach by experienced clinicians and infectious disease specialists is required in order to ensure the appropriate management of the syndrome itself, and the precipitating or underlying infection. Pediatricians should be alert and aware of the risk of the syndrome, because an early diagnosis can change its natural history and it has been shown that prompt treatment improves the overall prognosis. A combination of high fever unresponsive to broad-spectrum antibiotics, hyperferritinemia, hypertriglyceridemia, hypofibrinogenemia, cytopenia, organomegaly and characteristic histological findings in the setting of an infectious process (particularly EBV infection, but also other viral and bacterial, parasitic and fungal infections) is the key diagnostic clue. A better understanding of the pathophysiology of HPS should clarify the interactions between immune system pathways and infections. Specific antimicrobial therapy can be beneficial in selected cases, whereas antiviral drugs do not seem to be curative. Severe cases of infection-dependent HPS require immunosuppressants or chemotherapeutic agents, while bone marrow transplantation is the ultimate choice for persistent refractory cases.

## Summary

Hemophagocytic syndrome (HPS) can occur as a rare complication of various infections in children. Clonal proliferation of T lymphocytes with an excessive activation of macrophages can be triggered by different infectious agents, thus indicating that infection *per se* is involved in the pathogenetic mechanism of the process. A number of studies have demonstrated that HPS is frequently triggered by one of many different viral, bacterial, parasitic or fungal infections, with large differences in terms of treatment responses and overall outcomes. All patients meeting the criteria for HPS should undergo initial tests to diagnose the underlying infecting organism, which should be guided by epidemiological data and the patient’s medical history. A polymerase chain reaction search for infectious agents, including EBV, CMV and *Leishmania*, is recommended in a clinical scenario characterised by unremitting fever, organomegaly, cytopenia and hyperferritinemia. As HPS may be associated with many infectious diseases and immunological, neoplastic or genetic disorders, the close cooperation of pediatricians and infectious disease specialists is crucial in order to define any precipitating or underlying condition.

## Abbreviations

CMV: Cytomegalovirus; EBV: Epstein-Barr virus; HHV8: Human herpes virus 8; HIV: Human immunodeficiency virus; H5: Hemagglutinin; HPS: Hemophagocytic syndrome; NK: Natural killer.

## Competing interests

The authors declare that they have no competing interests.

## Authors’ contributions

VA and DR drafted the manuscript and contributed equally to its preparation; SE revised the draft and made a substantial scientific contribution. All of the authors read and approved the final version of the manuscript.

## Pre-publication history

The pre-publication history for this paper can be accessed here:

http://www.biomedcentral.com/1471-2334/13/15/prepub
